# From the reference human genome to human pangenome: Premise, promise and challenge

**DOI:** 10.3389/fgene.2022.1042550

**Published:** 2022-11-10

**Authors:** Vipin Singh, Shweta Pandey, Anshu Bhardwaj

**Affiliations:** ^1^ University Institute of Biotechnology, Chandigarh University, Mohali, India; ^2^ Academy of Scientific and Innovative Research (AcSIR), Ghaziabad, India; ^3^ Bioinformatics Centre, CSIR-Institute of Microbial Technology, Chandigarh, India

**Keywords:** human pangenome, reference human genome, graph based methods, variant discovery, precision medicine

## Abstract

The Reference Human Genome remains the single most important resource for mapping genetic variations and assessing their impact. However, it is monophasic, incomplete and not representative of the variation that exists in the population. Given the extent of ethno-geographic diversity and the consequent diversity in clinical manifestations of these variations, population specific references were developed overtime. The dramatically plummeting cost of sequencing whole genomes and the advent of third generation long range sequencers allowing accurate, error free, telomere-to-telomere assemblies of human genomes present us with a unique and unprecedented opportunity to develop a more composite standard reference consisting of a collection of multiple genomes that capture the maximal variation existing in the population, with the deepest annotation possible, enabling a realistic, reliable and actionable estimation of clinical significance of specific variations. The Human Pangenome Project thus is a logical next step promising a more accurate and global representation of genomic variations. The pangenome effort must be reciprocally complemented with precise variant discovery tools and exhaustive annotation to ensure unambiguous clinical assessment of the variant in ethno-geographical context. Here we discuss a broad roadmap, the challenges and way forward in developing a universal pangenome reference including data visualization techniques and integration of prior knowledge base in the new graph based architecture and tools to submit, compare, query, annotate and retrieve relevant information from the pangenomes. The biggest challenge, however, will be the ethical, legal and social implications and the training of human resource to the new reference paradigm.

## 1 Introduction

On 12 February 2001, The Human Genome Project Consortium announced the release of the first draft of the Reference Human Genome and the sequence was released into the public domain. Parallelly, Celera Genomics, a private initiative, also announced the release of the Alternate Human Genome assembly (Lander et al., 2001) (Venter et al., 2001). Considered as the “giant leap” in Biotechnology, this was an event no less celebrated than the landing of man on the moon, and divided modern Biotechnology into pre and post human genome era. This also marked the beginning of the Omics era - the study of something in totality and not in parts (Kiechle, Zhang, and Holland-Staley 2004). The Reference Human Genome remains the single most important resource for mapping human genetic variations and assessing their clinical impact. However, it was immediately realized that if we were to tap the full potential of the sequence information in terms of understanding genotype-phenotype correlation, mapping disease causing variations, in pharmacogenomics and in personalized medicine, a large number of individuals need to be sequenced in quick time and at an astronomically lower cost. The prohibitively high cost and time of sequencing genomes in 2001 lead researchers to explore alternate scale up technologies to Sanger Sequencing, so as to bring down the cost of sequencing to an affordable one thousand dollars. Technological advances in sequencing techniques, as also in information technology including high performance computing and development of novel algorithms lead to Second Generation short read sequencers. Given the short read lengths (100–400 bases depending on the sequencing platform) generated by the Second generation sequencers, accurate *de novo* assembly of the fragmented parts in large, complex and repeat rich genomes such as the human genome was improbable and a reference based assembly approach whereby the short reads were aligned and mapped on to the reference human genome was followed. Third generation long read sequencing techniques are now gaining attention as these, in combination with short read sequencers, allow almost error less *de novo* assembly of complex repeat rich genomes (Hu et al., 2021). As compared to three billion dollars and 10 years in 2001, a good quality, high coverage and accurate haplotype phased telomere-to-telomere assembly of the human genome can be obtained at about a 1000 dollars and in half a day today. Thus, technological advancements in data generation as well as analysis provide us with an opportune moment to gain further insights in human genetics and disease association.

In this article, we critically examine the limitations of the reference human genome and the need to redefine the reference *per se* - the human pangenome–a composite of multiple, haplotype resolved telomere-to-telomere assemblies. We assess the progress made in sequencing technologies since the release of the first draft of the reference human genome, which now make it possible to conceive the pan genome reference. We conclude with a discussion on the promises and challenges as we take definitive steps towards redefining the reference for human genetic studies.

### 1.1 The reference human genome and genomic variations

The working draft of the reference human genome was released in 2001 and the finished euchromatic genome was released in 2004 (International Human Genome Sequencing Consortium 2004) and has been revised several times since then. The current assembly GRCh38. p13/hg38 was released in December 2013. The reference human genome represents a linear coordinate system or grid facilitating the mapping and assembly of reads obtained from other sequencing experiments and serves as a standard for identifying the variations therein. It is the most extensively used resource for human medical genetics and genomics applications. Comparison of a human genome with the reference human genome allows identification of genomic variations which may associate with the observed phenotypes. Genomic variations have been studied extensively to understand their role in Mendelian and non-Mendelian disease association, diagnostics, prognostics, pharmacogenetics and pharmacogenomics. These genomic variations include the most commonly occurring–single nucleotide substitutions or Single Nucleotide Variations - SNVs, small (<50 base pairs) insertions/deletions, known as INDELS, large structural variations including large INDELS, segmental duplications - duplications of 1 kb or more, differences in copy numbers in tandem repeats, the presence/absence of transposon or mobile element insertions as well as large scale genomic rearrangements like translocations, inversions etc (Eichler 2019). A genomic variant occurring at a frequency of more than 1% in the population is referred to as polymorphic. Single Nucleotide Variation or Single Nucleotide Polymorphism is the most common type of variation found in the human genome. A typical genome differs from the reference human genome at 4.1 million to 5.0 million sites, suggesting that apart from the raw sequence data, one also needs to cater for 4.5 million variant sites if the comparison was done to reference human genome (Auton et al., 2015).

However, the reference human genome, which is used as the standard to elucidate the variations, is neither complete nor does it represent an exhaustive catalog of variations that may exist in the population. It represents a linear composite of merged haplotypes coming from predominantly European ancestry, with a single individual, of more than 20, contributing more than 70% of the reads used for the assembly (Ballouz, Dobin, and Gillis 2019). The reference human genome thus underrepresents and underestimates the full extent of variation that may exist in the population. In addition, due to limitations of read length offered by Sanger Sequencing technique, it is also not complete with gaps in centromeric, telomeric and other repeat rich regions. More than 50% of the gaps in the genome relate to complex Segmental Duplications. It is estimated that the use of short reads and reference based assembly may have resulted in non-reporting of more than 70% of the structural variations (Vollger et al., 2022). This results in a reference bias as well as an ascertainment bias confounding variant discovery, gene-disease association studies and inaccuracies in genetic analysis. The reference human genome is not ideally suited to serve as the “reference” (Chen et al., 2021).

## 2 Additional efforts to catalog and annotate human genomic variations

A need for exhaustive functional annotation of the genome was reflected in the ENCODE project (Dunham et al., 2012). A deeper cataloging of the variation that exists in the population was the motivation behind HapMap (Altshuler, Donnelly, and The International HapMap Consortium 2005), the 1000 genomes project and the 100000 genomes project besides others. The 1000 genomes Project reconstructed the genomes of 2,504 individuals from 26 populations using a combination of omics technologies including whole-genome sequencing at low coverage (average depth 7.4X), sequencing of the exome at high coverage (average depth 65.7X), and dense microarray genotyping and reported 84.7 million single nucleotide polymorphisms (SNPs), 3.6 million short insertions/deletions (indels), and 60,000 structural variants, all phased onto high-quality haplotypes. The structural variations catalogue comprises of 42,279 biallelic deletions, 6,025 biallelic duplications, 2,929 mCNVs (multi allelic copy-number variants), 786 inversions, 168 nuclear mitochondrial insertions (NUMTs), and 16,631 mobile element insertions (Auton et al., 2015).

**FIGURE 1:**
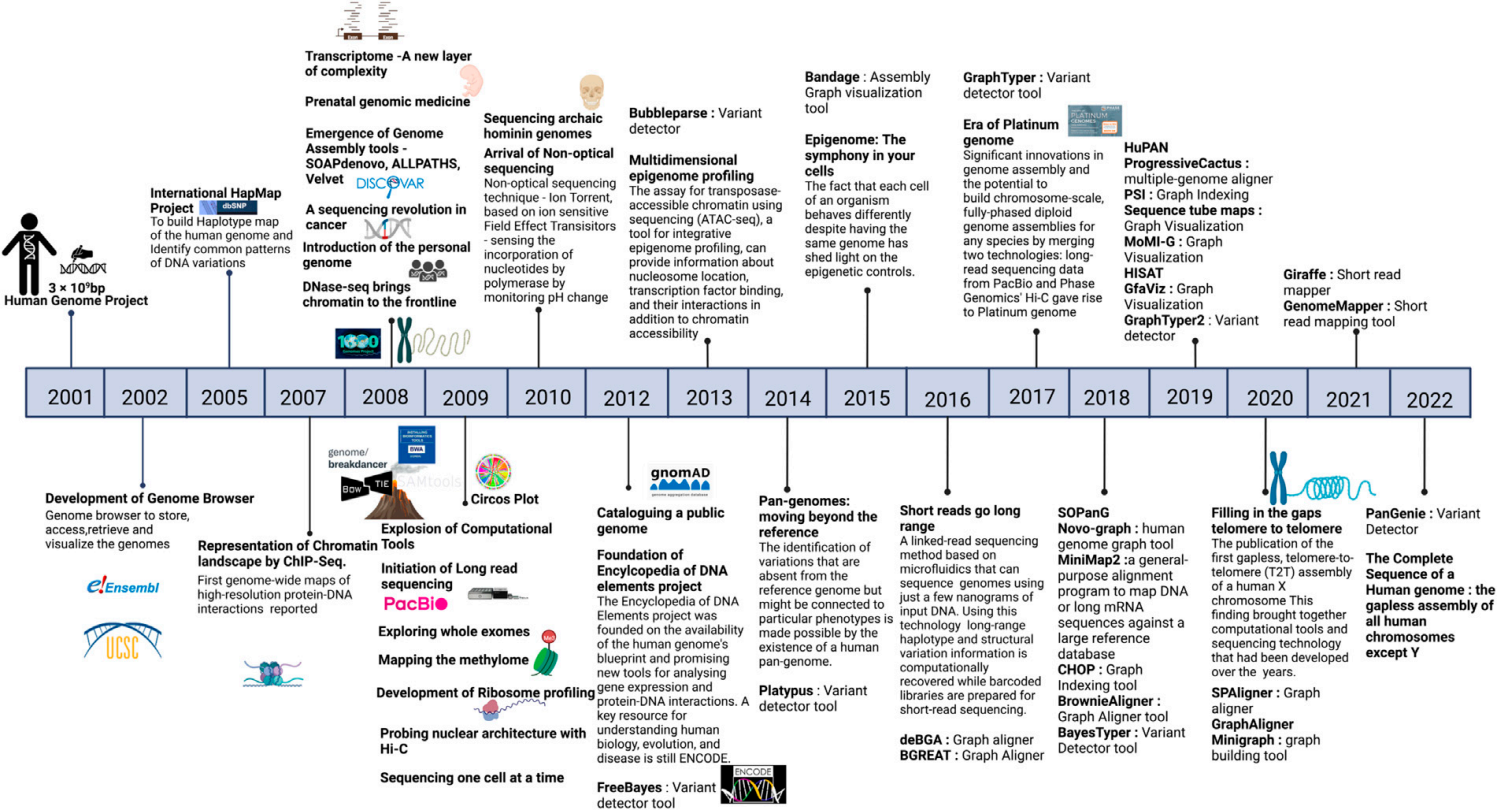
Timeline of major milestones in the last twenty years of human genomics. Created with BioRender.com.

**FIGURE 2:**
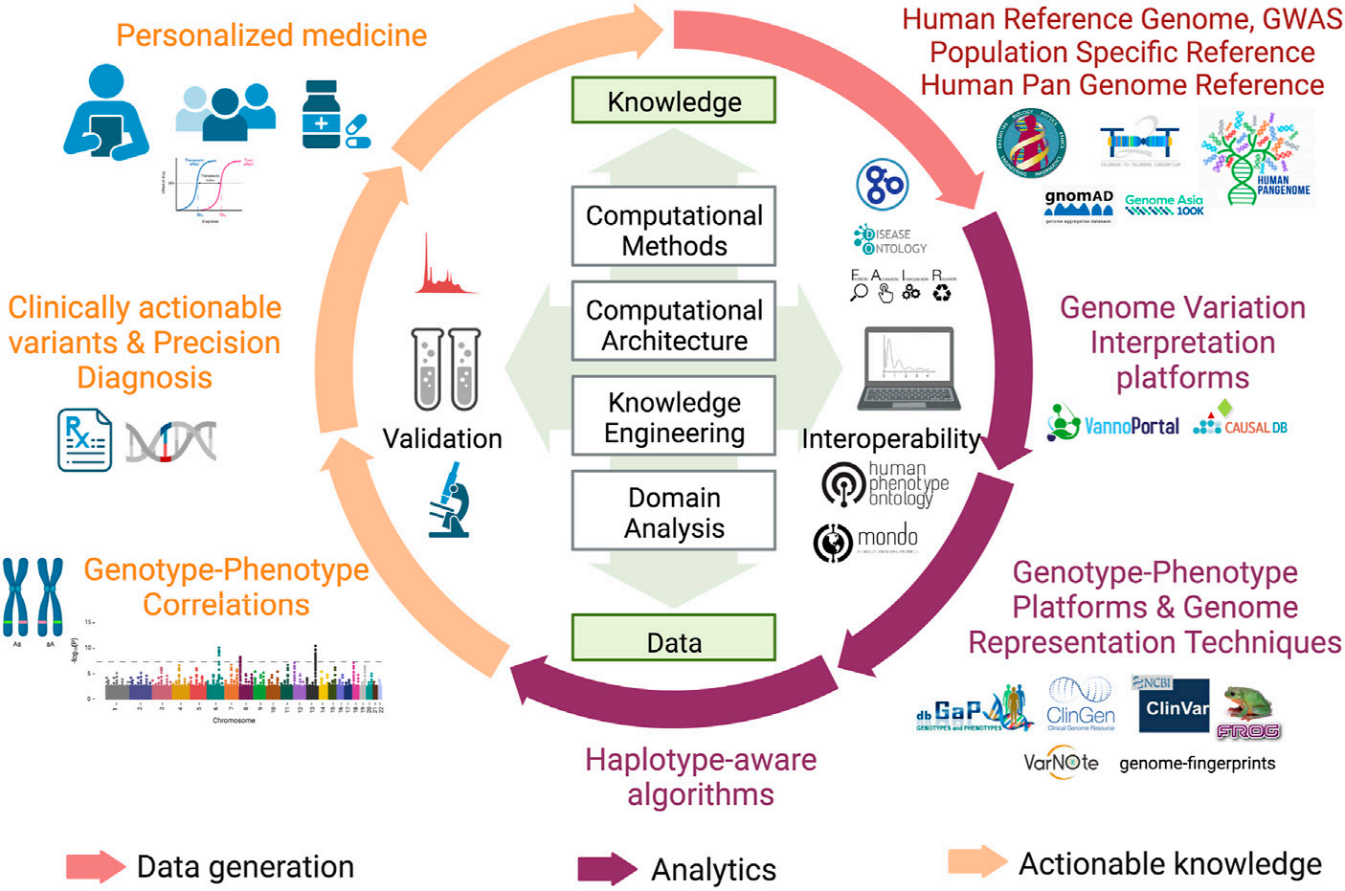
Overview of approaches for biomedical applications of human genome studies. Adapted from “The Clinical Applications of Translational Bioinformatics”, by BioRender.com (2022). Retrieved from https://app.biorender.com/biorender-templates.

With a decade of advancement in sequencing technologies that led to sequencing of a large number of genomes, the objective of the sequencing approach towards genetic screening or predisposition was further extended to target precision medicine. This extended vision demanded discovery and detailed annotation of disease associated variants including the rare variants, ushering in the era of population specific reference genomes. As more and more geographical regions sequenced indigenous populations, it became evident that the under-representation of the non-European samples in human genetic studies was limiting in capturing diversity of genomic datasets which significantly impact the clinical relevance of pathogenic variants identified in European samples to other datasets (Popejoy and Fullerton 2016). It was therefore realized that population specific reference genomes and Genome Wide Association Studies (GWAS) across diverse populations are required to capture the human genetic diversity which was otherwise missing and are critical to understanding disease biology. These efforts are also critical to annotate variants of unknown significance which were reported in large cohort studies but could not be discovered in underrepresented populations as well as in identifying false positive associations. Moving away from the persistent bias, several initiatives like the GenomeAsia100K project (Wall et al., 2019), H3Africa (Mulder et al., 2018), All of United States, IndiGenomes (Jain et al., 2021), etc were initiated to capture genetic variation, explore population structure, identify disease associations and map founder effects in diverse populations across the world. These projects also contributed to discovery of rare disease associated variants. A large number of such initiatives spawned overtime, making it imperative to aggregate these datasets for better understanding of population frequencies of variants, discover novel rare variants, identify novel disease associated genes and variants and prioritize the variants across different population groups. In this context, the Genome Aggregation Database (gnomAD) is the largest collection of harmonized population variation dataset (195,000 individuals as of now) (Gudmundsson et al., 2022). Based on the current version (v3) of gnomAD it is observed that on an average a single human exome carries 27 ± 13 novel unique coding variants. More importantly, the average of such unique novel variants vary across different population groups currently present in gnomAD with South Asians reported to have 38 ± 14 novel unique variants. It is proposed that this number is expected to be higher in population groups that are currently not well represented in gnomAD. Other large databases include NHLBI’s TOPMed-BRAVO (Taliun et al., 2021) and DiscovEHR datasets (Wall et al., 2019). Furthermore, it has been shown that size of the datasets ancestral diversity increases the chances of discovery of rare variant. In this context, it is strongly recommended that the pathogenic status of already annotated pathogenic variants in databases like ClinVar (Landrum et al., 2018) needs to be revisited. A timeline of major milestones in the last 20 years of human genomics is illustrated in Figure 1 and an overview of approaches for biomedical applications of human genome studies is depicted in Figure 2.

## 3 Advancement in sequencing techniques - The three generation of sequencers

One of the primary drivers in the massive increase in sequence data and generation of diploid, phased, high coverage accurate assemblies and complete genomes, the primary prerequisites for generating the pangenome reference, is the evolution of sequencing technologies. Sanger Sequencing Technique was the only automated sequencing technique available at the time when the Human Genome Project was conceived in late 1980s. Considering its limited parallelization and intermediate read length of ∼900 bases, and the size and complexity of the human genome (more than 50% repeat content), the Hierarchical shotgun sequencing strategy was devised by the Human Genome Project to ensure a reasonably accurate assembly (Lander et al., 2001). The success of Sanger technique in sequencing the human genome also proved to be its Waterloo as it exposed the limitations–three billion dollars and 10 years to generate the reference human genome. The quest for sequencing techniques that could sequence a human genome in less than a thousand dollars led to several novel approaches broadly classified as Second and Third Generation Sequencing techniques. The second generation sequencing techniques had shorter read lengths (∼100 bases) than Sanger Sequencing but this was compensated by their massively parallel sequencing capabilities resulting in high throughput and high coverage quality data. However, the short reads posed an assembly challenge necessitating the use of a reference genome for alignment and mapping of short reads to assemble them into full genomes (Alkan, Sajjadian, and Eichler 2011). The third generation sequencing techniques largely consist of long read sequencers with an average read length >10 kb and facilitate *de novo* assembly of genomes. More significantly, they also enable mapping of some of the most intractable extensively repeat rich regions of the genome, not sequenced before, thus allowing for filling of the gaps and telomere to telomere assembly. Another advantage of the third generation sequencing techniques is that, as opposed to both first and second generation sequencing, where a complex bisulphite treatment step was involved, they allow a direct readout of the epigenetic state of nucleotides (Amarasinghe et al., 2020).

A shift from short read sequencers to long read sequencers which facilitates a more accurate, and complete and unbiased *de novo* assembly of genomes, including the intractable repeat rich regions as well as regions with high GC content is imminent (Pollard et al., 2018). With the recent release of the first telomere to telomere assembly of the human genome, T2T-CHM 13v2.0, adding nearly 200 million base pairs of novel DNA sequences, including 99 genes likely to encode for proteins and nearly 2,000 candidate genes that need further investigation (Nurk et al., 2022), an update of existing knowledge bases and resources and re-evaluation of prior comparisons has become imperative. This is a humongous task given the size and heterogeneity of genomic data. As more such complete genomes become available, a near complete catalogue of genomic variations and their functional impact may be unraveled, necessitating a multiple reference comparison.

## 4 The human pangenome

The plummeting costs of whole genome sequencing and the potential of long read sequencers to deliver complete, accurate, phased diploid assemblies with epigenomic status provides us with the most opportune moment to conceive a more informative, sophisticated complete reference human genome–the human pangenome - a collective whole genome sequence of multiple individuals capturing the maximum possible diversity that exists in the population. Towards this, the Human Pangenome Reference Consortium (HPRC) aims to create a graph based telomere to telomere representation of the global genomic diversity, to replace the current monophasic, incomplete reference human genome (Wang et al., 2022). To ensure that the pangenome is a true global representation of variations that exist in the human population, one of the goals of the HPRC is to identify individuals from diverse ethnic and biogeographical backgrounds and generate at least 350 reference quality telomere to telomere haplotype phased human diploid genomes - i.e. 700 haplotypes using long range sequencing techniques, trio binning and the use of haplotype aware algorithms. Initially, for high quality long read sequencing, the HPRC has selected the individual cell lines in the 1000 genomes project which already offers a deep catalog of human variation from 26 populations (Auton et al., 2015). In disease context, the comparison may have to be done to several normal genomes so as to zoom into the variations responsible for the disease phenotype and subtract the silent ones. Pilot studies from China and Africa underline the importance of Pangenome studies in elucidating novel sequence and novel variations in the human genome.

The Chinese Pan Genome project used 486 deep-sequenced Han Chinese genomes and reports 276 Mbp of novel DNA sequences not reported in the reference human genome. The novel sequences belong to one of the two subcategories - individual-specific and shared common sequences. The common sequences, when used along with the reference human genome, improved the accuracy of mapping and variant calling (Li et al., 2021).

The African Pangenome Project used a deeply sequenced dataset of 910 individuals of African descent, to identify unique set DNA sequences present in the African population but not represented in the reference human genome. Unmapped reads to the human genome were assembled into contigs and re-examined (all-to-all comparisons), to derive unique sequences in the African population, not represented in the reference human genome. This dataset consists of 296,485,284 bp in 125,715 distinct contigs indicating that the African PanGenome is 10% larger than the reference human genome. The functional consequences of this extra sequence is under investigation. 387 of these novel contigs are found in 315 distinct protein coding genes (Sherman et al., 2019).

Recently, The Human Pangenome Reference Consortium (HPRC) published a first draft human pangenome reference. of 47 phased, diploid assemblies of genetically diverse individuals in bioRxiv. Covering more than 99% of the genome length, with an accuracy of more than 99%, the pangenome reports novel alleles at structurally complex loci, adds 119 million base pairs of euchromatic polymorphic sequence and 1,529 gene duplications relative to the existing reference, GRCh38. An additional 90 million base account for the structural variation (Liao et al., 2022).

### 4.1 Challenges in handling and evaluating large genomic datasets

Genome sequence data is poised to overtake the cumulative data on social media by 2025. The total number of individuals whose genome would be sequenced by then would be anywhere between 100 million to two billion. The data-storage demands for this alone could run to as much as 2–40 exabytes because the number of data that must be stored for a single genome is 30 times larger than the size of the genome itself. While the per-base cost of sequencing is dropping by about half every 5 months, the price of data storage falls by half every 14 months. Thus, it is evident that our capacity to generate data is going to far exceed our capacity to store and analyze this data indicating an imminent data management problem. Dealing with this data deluge in terms of storage, retrieval, analysis and exchange therefore indispensably requires novel, interoperable and scalable platforms (Stephens et al., 2015).

#### 4.1.1 Genome variation discovery, annotation and representation

Variant discovery has largely relied on pairwise alignment with the reference human genome. However, as has already been mentioned, the reference human genome fails to capture the full array of variations in human population due to sampling errors and sequencing technology limitations of the times. Over the last 2 decade, there has been a tremendous increase in whole genome sequencing projects *via* Next Generation Sequencing technologies which has led to standardization of methods for variant discovery and generation of an equally vast array of genomic variants (DePristo et al., 2011). The methods of variant discovery are now evolving from reference based read alignment to graph based methods for capturing the complete diversity in human pangenome (Paten et al., 2017). Whole genome multiple assembly alignments with graph based dense representation of variations in the pangenome will facilitate a comprehensive and exhaustive detection of variations. Annotation of genes and other genomic features like regulatory elements including promoter, CpG elements, enhancers, boundary elements and repeats etc. will have to be overlaid on the pangenome. It is proposed that the pangenome will have both NCBI RefSeq and EMBL-EBI’s Ensembl/Gencode gene set. In addition, other transcriptomics data will be mapped to individual haplotypes to improve the current annotation and identify novel genes. To understand how genomic variations influence genome function and the phenotype (genotype-phenotype correlation) experimental data from RNASeq, MethylC and ATACSeq experiments from major projects like Roadmap Epigenomics, ENCODE, 4DNucleome, IGVF etc (Wang et al., 2022) will also be integrated.

**FIGURE 3:**
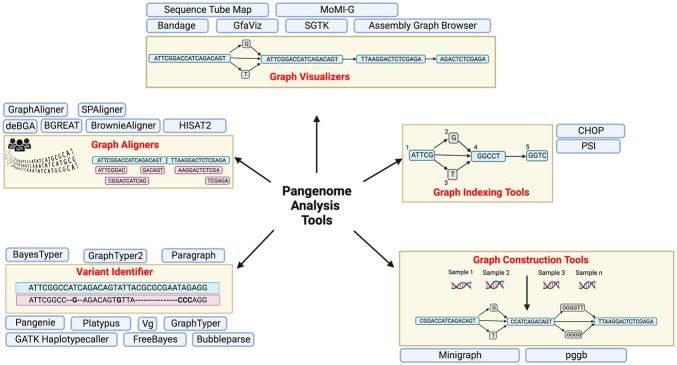
Pangenome applications and pipelines with brief description. Created with BioRender.com.

A whole new suite of user-friendly tools and analysis pipelines compatible with graph based architecture of the pangenome and maintaining organic continuity with the reference human genome will have to be developed for submission, alignment, visualization, analysis, format conversion, annotation and variant detection and sharing and retrieval of data. Some of these tools and pipelines already exist and will be improved overtime. These include graph building tools like minigraph (Li, Feng, and Chu 2020), graph aligners like deBGA (Liu et al., 2016), BGREAT (Limasset et al., 2016), HISTA2 (Kim et al., 2019) etc, tools for graph visualization including Bandage (Wick et al., 2015), AGB (Mikheenko, 2019) etc. and variant detection tools like PanGenie (Ebler et al., 2022), BayesTyper (Sibbesen et al., 2018), Paragraph (Chen et al., 2019), etc (listed in Table 1 and illustrated in Figure 3).

**TABLE 1:** List of pangenome applications and pipelines with brief description.

Tools	Description & variant identify	Year
FreeBayes Garrison and Marth (2012)	Identifies small variations - SNPs, MNPs, and INDELs (<50 bases). Uses the BAM files and reference genome as input. Detects haplotypes using Bayesian statistics	2012
Bubbleparse Leggett et al. (2013)	Uses Next Generation Sequencing (NGS) data to detect SNPs, without using the reference sequence	2013
Platypus Rimmer et al. (2014)	Detects small variations. Candidate variants are computed from read alignments, local *de novo* assembly, followed by local realignment and probabilistic haplotype estimation	2014
Bandage Wick et al. (2015)	Graph visualizer, the user can customize the graph by moving nodes, adding labels, altering colours, and extracting sequences while zooming in on particular regions of the graph. Does not support scaffold graphs	2015
deBGA Liu et al. (2016)	A graph-based aligner using the seed and extension approach. It organizes and indexes one or more reference genomes using de Bruijn graph (RdBG), and then aligns high-throughput sequencing (HTS) reads to those genomes	2016
BGREAT Limasset et al. (2016)	Assembly tool, uses a heuristic technique to map reads onto the branching path of de Bruijn graph (DBG)	2016
Graphtyper Eggertsson, (2017)	Used for identifying and genotype variations. Takes the reference genome as well as a list of known sequence variants in variant-call format (VCF) format as input, to construct a variant-aware pangenome graph. The seed-and-extend approach is then implemented to the read alignment	2017
BayesTyper Sibbesen, Maretty, and Krogh (2018)	A k-mer approach method that uses reference genome, sequence reads as well as the variant database of the candidate as input and constructs the variation graph. It genotypes all categories of variations (SNPs, indels and complex structural variants)	2018
BrownieAligner Heydari et al. (2018)	Alignment tool, uses seed and extend strategy to align short reads from Illumina to De Bruijn graph. Using high order Markov-model, it also resolves repeats in the graph	2018
GraphTyper2 Eggertsson et al. (2019)	Large Scale (tens of thousands of whole-genomes) identification of structural variants and small variants using pangenome graphs	2019
Paragraph Chen et al. (2019)	It is the graph-based genotyper that uses sequence graph for modeling structural variants (SVs) using short read sequence data	2019
HISAT2 Kim et al. (2019)	Quick and accurate algorithm used to align NGS data - DNA/RNA, to multiple human genomes and reference genome. It uses collection of small Graph FM (GFM) indexes that represent genome and with several alignment approaches, provides rapid alignment of reads	2019
GfaViz Gonnella, Niehus, and Kurtz (2019)	Visualization of sequence graph in graphical fragment assembly (GFA) format. Both command line and graphical user interface	2019
SGTK Kunyavskaya and Prjibelski (2019)	Enables the building and visualising the scaffold graphs using sequencing data	2019
Assembly Graph Browser Mikheenko (2019)	Visualization tool for large and complex assembly graph. Also helps in analysis of repeats and construction of assembly graph	2019
Sequence tube Map Beyer et al. (2019)	Visualization tool of genome graphs and displays variant information in tube format	2019
MoMI-G Yokoyama et al. (2019)	Web based visualization tool for genome graphs, identifies structural variants and hence useful for long reads analysis	2019
vgtoolkit Hickey et al. (2020)	It is used for SV genotyping by building variation graphs using either variant catalogs in the VCF format or assembly alignments	2020
GraphAligner Rautiainen and Marschall (2020)	Alignment tool for long reads. It supports both the GFA and variation graph (vg) formats and can operate with a variety of graphs, including those with overlapping and non-overlapping node sequences	2020
SPAligner Dvorkina et al. (2020)	It is multipurpose tool. It aligns nucleotide and protein sequence to assembly graph and effectively align reads from third generation sequencing	2020
CHOP Mokveld et al. (2020)	Indexing tool for population-graph that uses haplotype-level data to limit path indexing without requiring any pruning or heuristic filtering stages. This constrain eliminates the requirement to assess all k-paths	2020
Minigraph Li, Feng, and Chu (2020)	A sequence-to-graph mapper, that constructs the pangenome graphs using haplotype data and a minimap2 -like algorithm which is based on the seed-chain-align procedure	2020
GenomeMapper https://1001genomes.org/software/genomemapper.html	Short read mapper which align short reads either to reference genome or multiple genomes	2021
Pangenie Ebler et al. (2022)	It infers the genotype of the sample by taking a pangenome graph and short-read sequencing data as input and integrates the information with k-mer count using hidden Markov model (HMM). It enables the variants analysis of SNPs, INDELs, and SVs	2022
pggb https://github.com/pangenome/pggb	Toolkit to build the pangenome graph *via* the integration of various packages. Wfmash is used for pairwise sequence alignment, sequish is used for graph induction, and smoothxg and gfaffix are used for normalization of graph. Visualization can be done using Optimized Dynamic Genome/Graph Implementation (ODGI)	2022
ODGI Guarracino et al. (2022)	Toolkit that helps in the understanding of pangenome graphs. Provides tools for identifying complex regions within pan-genomic loci, and analyzing, manipulating, and visualizing the pangenome graph at the gigabase scale	2022

Assessing the clinical impact of genomic variations in the context of disease association is the ultimate goal of human genetic studies. Several annotation features like allele frequency, tissue or cell type specificity, phenotype association, heterozygosity, functional impact, etc are important to understand the context-dependent significance of genomic variations. Towards this, platforms like VannoPortal (Huang et al., 2022), CausalDB (Wang et al., 2020), DisGenNet (Piñero et al., 2017), PhenGenVar (Shin et al., 2022), etc have been developed. Data from these and other platforms can be utilized by disease-gene network resources like HumanNet v3 for exploring mechanistic insights (Kim et al., 2022). The true potential of these platforms lies in the fact that these variants can be compared across several individuals in a variety of different phenotypes. However, given the complexity of size and data representation methods, it becomes a daunting task to perform these analyses at scale and demand novel methods of data representation towards scalable data analysis.

A few groups have made attempts to develop novel data representation techniques in the form of fingerprints so as to simplify the task of comparison both from a computational as well as biological perspective. These tools include Fingerprinting Ontology of Genomic variation (FROG) (Abinaya, Narang, and Bhardwaj 2015), Ultrafast method (Glusman et al., 2017), Bitome (Lamoureux et al., 2020), VarNote (Huang et al., 2020), etc. To the best of our knowledge, FROG is the first method published in this direction and utilizes an ontology-based approach for representing genomic variation. FROG not only represents variation but also provides a comprehensive assessment of the impact of the variation at the levels of chromosome, DNA, RNA, protein or their interactions. Moreover, FROG ontologies are not dependent on genome data size, organism or on the diversity of ways in which impact of SNPs are reported. It also represents data in binary format to generate genome variation fingerprints for efficient computation, data compression and reducing dimensionality for comparison of the same across several individuals or populations. The Ultrafast method considers the position of the reference and the alternate alleles in an individual and applies a method of locality sensitive hashing for representation of genomic variants with the primary objective of landscaping population structure and not with the objective of variation interpretation. In fact, the method does not allow the variants to be traced back, making it a genome representation method suitable for managing datasets where privacy is a matter of concern. However, given the lack of variant interpretation aspects, this method is not suitable for data representation for genotype-phenotype correlations. Bitome, a method primarily developed to study prokaryotic genomes, represents genomic features at the level of base pairs and is shown to provide an overall profile of genomic features distributed across the genomes. One of the most recent methods, VarNote, performs rapid annotation of genome-scale variants and has been shown to prioritize causal regulatory variants for common diseases. This method utilizes parallel random-sweep searching algorithm and a novel indexing system for the same. However, all these methods are yet to be customized and developed to capture the complexity of the human pangenome and represent the impact of genome-wide variation in disease association studies.

## 5 Discussion

The current reference human genome assembly serves as a linear, coordinate system for sequence comparisons. While this is useful, differences from the reference are difficult to observe and, except for SNPs, confounding to describe by virtue of being exclusively present or absent in the reference.

Pangenomic methods allow all-to-all comparisons of multiple genomes and derive relations to each-other in the form of a pangenome graph. In this graph, sequences and variations therein are merged into a single coherent data structure. While still undergoing improvisations, broad parameters of sequence graph model, and the input and output data formats are reasonably well defined. Graphical Pangenome are usually represented in Graphical Fragment Assembly format (GFA). Graph Nodes are stored in sequence records (S), edges represented as link (L) records, and embedded sequences in path records. Mappings to GFA can be encoded in GAM (Graph Alignment Map format) or text based Graph Alignment Format (GAF).

Several Pangenome Graph tools for alignment, graph construction and genotyping of small (less than 50 bases–SNP, MNP and small INDELS), medium and large variations (structural variations- >1kb, inversions, balanced translocations, repeat polymorphisms etc) are already available and improvements and improvisations in terms of sensitivity, speed, space and memory utilization with emphasis on scaleup are ongoing. Some of these tools include the Pan Genome Graph Builder (pggb)–a pan genome Graph construction pipeline to create a pangenome graph of multiple genome sequences (https://github.com/pangenome/pggb), the variation graph toolkit -vg, a collection of computational methods for efficient mapping of reads on variation graphs using generalized compressed suffix arrays (Hickey et al., 2020). Assembly and graph visualization tools are also available - these include MoMI-G or Modular Multi-scale Integrated Genome graph browser (Yokoyama et al., 2019), GraphAligner (Rautiainen and Marschall 2020), Pantograph (Chen et al., 2019) and GfaViz (Gonnella, Niehus, and Kurtz 2019), Sequence Tube Map (Beyer et al., 2019), Bandage (Wick et al., 2015) etc. To ensure quality control, Pan Genome Graph Evaluator selects the best pangenome graph construction (https://github.com/pangenome/pgge). The compression of graph data can be achieved through GWBT which is based on Burrows-Wheeler Transform (https://github.com/jltsiren/gbwt).

### 5.1 Technical challenges

As discussed, a decent start and steady progress has been made with respect to generation, visualization and analysis of Pangenome data and is reflected in multiple aligners, graph generation, indexing and visualization tools (Table 1; Figure 3). Nevertheless, scale up remains the biggest challenge, and as more personal genomes data becomes available, the size of the beginning dataset -thousands of gigabse-scale genomes, is only going to grow exponentially demanding further time, memory and space efficient analysis algorithms and data representation formats.

These methods should not only cater to the linear reference genome, which has been at the core of reporting genomic variations, but also to the emerging datasets from the Human Pangenome studies. It is also important to mention here that standard ontologies are imperative for data interoperability and comparative genomics at scale and therefore the platforms thus developed should have built-in features for the same. Variation barcoding methods are recommended to represent genomic signatures both in the coding and the repeats regions of the genome. These barcodes may facilitate efficient comparison of genome-wide differences in the personal genomes (and also to human pangenome) making them more amenable for downstream analysis. The genomic signatures can be represented at various levels including the number of sites with specific signature, their location, functional annotation and distribution. It is imperative that genome-wide data is viewed at multiple-scales for better comprehension. Towards this, it is proposed that the data generated and analysed in the process may be coded as binary fingerprints, which not only needs less storage space but also makes the retrieval, analysis and sharing more scalable. Such platforms should also have features to annotate various genic signatures, identification of pathogenic variants and several repeat-associated genomic rearrangement signatures including but not limited to target site duplications, 3′ and 5′ flank transductions, insertion-mediated deletion, recombination mediated deletions, etc. (Singh and Mishra 2010). Towards this end, we propose a Personal Genomics Signature Platform (PGSP) - a standard ontology based platform to organize and classify the variation data and develop a universally applicable memory efficient language independent binary fingerprint for all variations that exist in the human genome. The Binary code allotted to each variant with respect to reference human pangenome is expected to facilitate easy classification, storage, retrieval and comparison of such variations across platforms. It will not only allow for data storage more efficiently but will also facilitate data interoperability. The Personal Genomics Signature Platform (PGSP) will allow for detailed annotation of the variation. This platform will also facilitate identification of genome-wide signatures among individual genomes. The algorithms thus generated will be applicable for better understanding of the role of genomic variations in inter-individual differences towards disease predisposition and drug-responses. PGSP should be developed as a universal, language independent, scalable binary digits based ontology for understanding the complex genotype-phenotype associations. The binary fingerprinting is likely to facilitate creation of a modular Minimal Code with Maximum Information (MCMI), shareable across different platforms and languages.

### 5.2 Ethical, legal, and social implications

As the data set expands from the currently proposed set of 700 haplotypes (350 individuals), one of the major challenges would be to ensure inclusivity. Linguistic, literacy, socio-economic barriers, coupled with the feeling of distrust among the racial-ethinc minorities and the aborigines restrict inclusivity in such projects (Dodson and Williamson 1999), Informed consent of participants requires that the participant be adequately educated about the project and its implications, which is a challenge in itself. Data privacy and protection in the era of open science only add to the ethical and legal complexity–the subjects need to be made consciously aware of how practices such as open science, data sharing and maintenance of electronic health records may impact their data and pose risks to privacy (Couzin-Frankel 2010). Lastly, the extent of information to be released to the subject, post the analysis and annotation of genomic data constitutes another layer of ethical dilemma. Legally, the subject is liable to complete information, but the impact this complete information and its interpretation can have on the subject’s personal mental health, of the family and societal attitude towards the subjects provides room for reasonable constraints.

Fully aware of the challenges ahead, the HPRC is armed with a team of ELSI scholars working at the interface of genomics, biomedical ethics, law, social sciences, demography and community engagement. The HPRC is mobilizing Indigenous geneticists, leaders and community members for the outreach programs to ensure development of an authentic and truly representative global reference resource, guided by the FAIR and CARE principles (Carroll et al., 2021)

The scientific challenge of ensuring a transition of a whole generation of researchers from the conventional linear coordinate system based on the reference human genome to a graph-based system is daunting in itself and would demand massive outreach programs *via* physical and online workshops, development of SOPs and user-friendly GUIs.

## 6 Conclusion

To conclude, the first step towards a better understanding and interpretation of new genome data is to replace the reference human genome–a nonrepresentative, monolithic, monophasic, incomplete standard by a human pangenome–a more accurate, inclusive, representative and complete composite of high-quality multiple telomere to telomere assemblies, maximally capturing the variations that exist in the human population. This would also entail novel representation methods, a new data structure - a graph based architecture and downstream suite of tools to submit, query, retrieve and analyze the pangenome efficiently towards meaningful inferences in a time, memory and cost efficient manner. Built-in interoperability of these platforms must also be ensured so that data from one platform can easily be imported and directly input to the next pipeline facilitating comprehensive evaluation of the inter-individual genomic variations and their functional and clinical significance. As discussed, the biggest challenge, however, will be the ethical, legal and social implications and the training of human resource to the new reference paradigm.

## Data Availability

The original contributions presented in the study are included in the article/supplementary material, further inquiries can be directed to the corresponding author.
